# The association between monocyte HDL ratio and albuminuria in diabetic nephropathy

**DOI:** 10.12669/pjms.37.4.3882

**Published:** 2021

**Authors:** Fatma Kaplan Efe

**Affiliations:** 1 Fatma Kaplan Efe, Department of Internal Medicine, University of Health Sciences Kecioren Research and Training Hospital, Ankara, Turkey

**Keywords:** Albuminuria, Diabetic nephropathy, Monocyte-to-HDL ratio

## Abstract

**Objectives:**

We aimed to investigate whether Monocyte-to-HDL ratio (MHR) had an association with albuminuria in patients with diabetic nephropathy (DN).

**Methods:**

Diabetic patients, who had admitted to the outpatient clinic of general internal disease department between September 2017 - February 2018 and had their spot urinary albumin/creatinine ratio measured, were examined retrospectively. Patients were separated based on the presence of DN. Patients with DN were grouped as Stage-I, Stage-II and Stage-III chronic kidney disease (CKD). Groups were compared in terms of MHR. The presence of a correlation between MHR and albuminuria was investigated.

**Results:**

MHR was found to be higher in the DN (n=85) group compared to Non- DN group. (16.2±5.5 vs. 14.3±4, p=0.037) And there was no significant difference in Stage-I, Stage-II and Stage-III CKD groups in terms of MHR. (15.2± 3.4, 16.1±6.0, 17.1±6.0, p=0.485). No significant correlation was found between MHR and albuminuria in DN and non-DN groups (p=0.634, r=0.052; p=0.553, r=-0.059).

**Conclusions:**

DN group had higher MHR than non-nephropathy group, whereas, there was no correlation between albuminuria and MHR.

## INTRODUCTION

Diabetic Nephropathy (DN) is the most frequent cause leading to renal failure.^1^ Microalbuminuria is the earliest finding of DN and is an indicator for cardiovascular mortality and morbidity in diabetic patients. Early diagnosis of microalbuminuria is important for preventing the progression of nephropathy. Urinary albumin/creatinine ratio must be measured for microalbuminuria screening.

Hyperglycaemia, polyols, advanced glycosylation end products and ischemia are effective in the pathogenesis of DN.^2^ The increased formation of advanced glycation end products leads to microvascular and macrovascular complications by causing endothelium and monocyte activation via pro-inflammatory effect. Recent studies have reported that monocyte/high-density lipoprotein (HDL) ratio (MHR) can be an indicator for inflammation and oxidative stress and that it can be used for cardiovascular diseases. Decreased serum HDL concentration and elevated monocyte count in the circulation have been shown in patients with chronic kidney disease. In our study, we aimed to determine the association between MHR and albuminuria in patients with DN and to evaluate the usability of the MHR for nephropathy.

## METHODS

Diabetic patients, who were admitted to the Internal Medicine outpatient clinic from September 2017 - February 2018 and had their spot urinary albumin/creatinine ration measured, were examined retrospectively. Patients were separated based on the presence of DN. DN was defined as spot urinary albumin/creatinine ratio ≥ 30 and/or eGFR < 60 mL/min. Patients with DN were grouped as Stage-I, Stage-II and Stage-III CKD. Stage-I CKD was defined as eGFR ≥ 90 mL/min and ACR ≥ 30; Stage-II CKD as eGFR=60-90 mL/min and ACR ≥ 30, and Stage-III CKD as eGFR < 60 mL/min. Total of 190 diabetic patients were included in the study. Ethics committee approval was received from the Health Sciences University Keçiören Education and Research Hospital at 09.09.2020 (Number: 2012-KAEK-15/2164).

Demographic and laboratory characteristics (age, sex, presence of hypertension, serum creatinine, eGFR, HbA1c, lipid profile, monocyte percentage, spot urinary albumine/creatinine ratio) of the patients were obtained from their medical records. eGFR was calculated based on 4-variable MDRD equation. Monocyte/HDL ratio (MHR) was calculated as (Monocyte percentage/HDL) x100.

Diabetes mellitus was defined as antidiabetic medication use or HbA1c ≥ 6.5. SPSS 21 software was used for statistical analyses. Continuous variables are presented as mean ± standard deviation; discrete variables are presented as %. Mann Whitney U test was used for continuous variables in the comparison of diabetic nephropathy and non-DN groups. Kruskal Wallis test was used for continuous variables in the comparison of patients with Stage-I, Stage-II and Stage-III chronic kidney disease. Chi-square test was used for the comparison of discrete variables. Spearman’s correlation coefficient was used for correlation comparison. p<0.05 was considered as statistically significant.

## RESULTS

**Fig.1:**
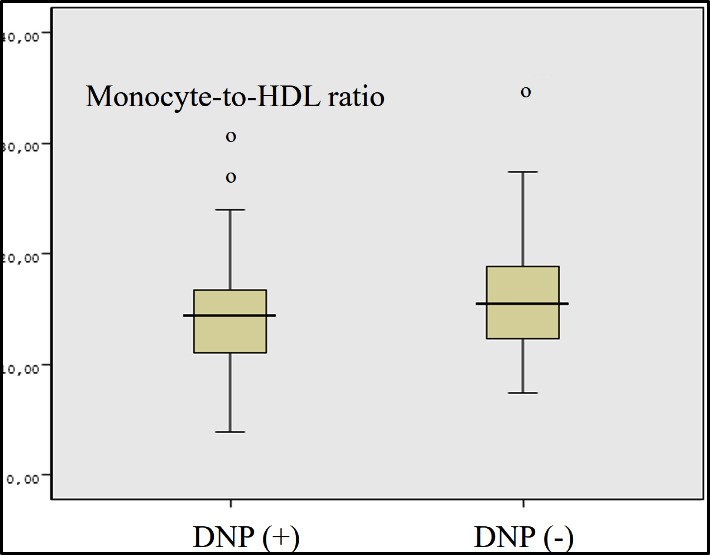
Monocyte-to-HDL ratio in in patients with diabetic nephropathy and without diabetic nephropathy (DNP; diabetic nephropathy.

Eighty-five patients with DN and 105 patients without DN were included in the study. Demographic and laboratory characteristics of DN and non-DN groups are shown in Table-I. MHR was determined to be significantly higher in DN group than the non-DN group (Fig.1).

**Table-I:** Comparison of demographic characteristics and laboratory findings of patients with and without diabetic nephropathy.

	Without diabetic nephropathy N=105	With diabetic nephropathy N=85	p
Age (year)	58± 10	63±10	0.210
Sex (F,%)	75(%71.4)	53(%62.4)	0.214
Cre (mg/dl)	0.76±0.09	0.98±0.25	<0.001*
eGFR (mg/dl)	91.7±11.6	72.2±21.1	<0.001*
ACR	11.7±5.9	143.8±170.3	<0.001*
HbA1c	7.6±1.8	8.3±2.2	0.033
Total cholesterol	201±39	206±41	0.464
LDL cholesterol	120±34	120±35	0.906
HDL cholesterol	48±11	46±10	0.362
Triglyceride	168±73	204±170	0.229
MHR	14.3±4.5	16.2±5.5	0.037*
Hypertension (%)	%51.4	%83.5	<0.001*

Classification of DN patients based on their CKD stages was found to be 22.4%, 43.5% and 34.1% for Stage-I, Stage-II and Stage-III, respectively. There was no significant difference in terms of MHR among patients with Stage-I, Stage-II and Stage-III chronic kidney disease (15.2±3.4, 16.1±6.0, 17.1±6.0, p=0.485).

No significant correlation was found between MHR and albuminuria in DN and non-DN groups (p=0.634, r=0.052; p=0.553, r=-0.059). There was no significant correlation between MHR and eGFR in DN and non-DN groups (p=0.218, r=-0.135; p=0.118, r=0.153). Hypertension frequency was higher in the DN group (83.5% vs 51.4%). No significant difference was found between mean MHR values of patients with and without hypertension (15.3±5.2 vs 14.9±4.9, p = 0.701).

## DISCUSSION

In this study, DN patients were found to have higher MHR value. However, no correlation was found between albuminuria and MHR. In recent years, diabetes is the most important disease-causing renal failure. Earliest indicator of DN is the presence of albuminuria. Albuminuria is a predictor for renal functions. As regression of microalbuminuria is possible through treatment, microalbuminuria screening is very important to prevent progression to renal failure and to determine cardiovascular disease risk.^3^,^4^ Spot urinary albumin/creatinine ratio measurement is performed for screening test.^5^ Due to the difficulties involved in the collection of 24-hour urine samples, using these samples for screening is not a practical method. Albuminuria increases in cases of urinary infection, uncontrolled hypertension, high intensity exercise, cardiac insufficiency, excessive protein intake and diabetic coma.^5^ Albuminuria is classified as normal if <30, as microalbuminuria between 30-300, and as macroalbuminuria if >300.^6^ Type-1 DM patients should undergo screening five years after the onset of diabetes, and Type 2 DM patients starting from the time of diagnosis.^7^

Two mechanisms are effective in the pathogenesis of DN. With the first mechanism; hemodynamic changes occur in glomerular membrane, tubular basal membrane, Bowman capsule thickens, tubulointerstitial renal fibrosis and glomerular filtration rate (GFR) decreases.^8^-^9^ And with the second mechanism, aldose reductase enzyme activation is induced via glucotoxicity polyol pathway and glucose is converted to sorbitol, and advanced glycation end products (AGE) are formed as a result of numerous biochemical mechanisms. AGE, oxidized lipids, free oxygen radicals, free fatty acids trigger inflammation. AGE receptors are present in macrophages, endothelium cells and mesenchymal cells. Inflammatory cytokine release from these cells induce adhesion molecule-mediated VCAM, ICAM monocyte activation and inflammatory cytokines (IL-1, IL-6, IL-18, CRP, TNF alpha etc.) are released Monocyte activation leads to chronic inflammation and atherosclerosis.^10^ These alterations cause changes in renal hemodynamics, GFR and blood pressure.^11^

Monocytes play a role in atherosclerosis with their pro-inflammatory and pro-oxidant effects.^12^-^13^ Macrophages are differentiated from circulating monocytes. In atherosclerosis, modified low-density lipoproteins (LDL) are captured by macrophages, forming cholesteryl ester loaded plaques by settling in intima and subintima. Circulatory monocyte count is a predictor for new plaque formation.^14^ HDL-C blocks the pro-inflammatory and pro-oxidant effects of monocytes by preventing macrophages migration and LDL oxidation.^15^ Anti atherosclerotic effect of HDL is mainly based on its effect on reverse cholesterol transport. At the same time, HDL-C suppresses the monocyte activation and macrophage proliferation. HDL-C causes vasodilation by increasing endothelial nitric oxide synthesis.^16^ Therefore, monocyte accumulation and HDL-C decrease leads to atherosclerosis and cardiovascular disease. Studies have found high monocyte count and low HDL-C levels to be signs of inflammation and atherosclerosis.^17^-^18^ The relation between these two parameters provides better understanding for inflammation.

In a study by Ganda et al. elevated monocyte count and increased atherosclerosis was detected in patients with mild renal dysfunction.^19^ These patients have decrease in HDL-C level and increase in both monocyte count and its activation. MHR increase parallel to decreased eGFR has been shown.^20^ Karatas et al. reported that MHR was positively correlated to the urinary albumin / creatinine ratio, independent of all other variables.^21^ Similarly, Kahraman et al. reported that MHR was positively correlated to 24-hour urinary albumin excretion and negatively correlated to GFR.^22^ Two different studies have shown that MHR is an independent predictor for contrast-induced nephropathy.^23^,^24^ In our study, although there was no significant difference in MHR value of patients with Stage-I, Stage-II and Stage-III chronic kidney disease, MHR was found to increase with CKD stage. Due to the few number of patients, this increment may not have reached statistical significance. In a study conducted by Kanbay et. al. MHR was shown to increase with decreasing renal function in predialysis renal patients.^17^ In addition, authors also reported that MHR can be evaluated as an independent risk factor for its association with poor cardiovascular profile and major cardiovascular event occurrence.^25^ Similarly, Cetin et al. stated that MHR can be used as an independent determinant for the severity of coronary artery disease and the possibility of future cardiovascular events in patients with acute coronary syndrome.^18^

Hypertension is another disease that may be associated with nephropathy. Aydin et al. found that MHR was higher in patients with primary hypertension and asymptomatic end-organ damage. 26 In the same study, proteinuria was considered as asymptomatic end-organ damage. In a comprehensive meta-analysis, Wagnew et al. reported that diabetic nephropathy is more common in diabetic patients accompanied by hypertension.^27^ It has also been suggested in different studies that the accompanying hypertension significantly contribute the nephropathy that occurs in diabetic patients.^28^,^29^ Similarly in our study, MHR was higher in patients with diabetic nephropathy when compared to the diabetic patients without nephropathy. It has been suggested that the coexistence of these two common diseases causes an increase in oxidative stress and inflammation, and the addition of hypertension to diabetes mellitus contributes to nephropathy by increasing glomerular pressure.^30^,^31^ The cause-effect relationship could not be examined due to the design of our study. The different frequency of hypertension between groups with and without nephropathy is consistent with previous studies and is an expected finding. However, it should be noted that the fact that the relationship between MHR and hypertension was not examined in our study is an important limitation.

### Limitation of the Study

The limitation of this study was the few number of patients and the failure to obtain results about the cause-and-effect relation between DN and high MHR levels due its sectional study design.

## CONCLUSION

In conclusion, DN patients were found to have higher MHR value. However, there was no significant correlation between albuminuria and MHR. Comprehensive studies are required to reveal the association between MHR and albuminuria.

### Authors Contribution:

**FKE** conceived, designed and did statistical analysis, editing of manuscript, collection and manuscript writing and final approval of manuscript.
